# Immunization Coverage and Antibody Retention against Rabies in Domestic Dogs in Lusaka District, Zambia

**DOI:** 10.3390/pathogens10060738

**Published:** 2021-06-11

**Authors:** Chiho Kaneko, Michihito Sasaki, Ryosuke Omori, Ryo Nakao, Chikako Kataoka-Nakamura, Ladslav Moonga, Joseph Ndebe, Walter Muleya, Edgar Simulundu, Bernard M. Hang’ombe, George Dautu, Masahiro Kajihara, Akina Mori-Kajihara, Yongjin Qiu, Naoto Ito, Herman M. Chambaro, Chihiro Sugimoto, Hideaki Higashi, Ayato Takada, Hirofumi Sawa, Aaron S. Mweene, Norikazu Isoda

**Affiliations:** 1 Unit of Risk Analysis and Management, Hokkaido University International Institute for Zoonosis Control, North 20, West 10, Kita-ku, Sapporo 001-0020, Hokkaido, Japan; ckaneko@cc.miyazaki-u.ac.jp (C.K.); cnakamura@mail.biken.or.jp (C.K.-N.); 2 Division of Molecular Pathobiology, Hokkaido University International Institute for Zoonosis Control, North 20, West 10, Kita-ku, Sapporo 001-0020, Hokkaido, Japan; m-sasaki@czc.hokudai.ac.jp (M.S.); hermcham@gmail.com (H.M.C.); h-sawa@czc.hokudai.ac.jp (H.S.); 3 Division of Bioinformatics, Hokkaido University International Institute for Zoonosis Control, North 20, West 10, Kita-ku, Sapporo 001-0020, Hokkaido, Japan; omori@czc.hokudai.ac.jp; 4 Laboratory of Parasitology, Faculty of Veterinary Medicine, Graduate School of Infectious Diseases, Hokkaido University, North 18, West 9, Kita-ku, Sapporo 060-0818, Hokkaido, Japan; ryo.nakao@vetmed.hokudai.ac.jp; 5 Department of Para-Clinical Studies, School of Veterinary Medicine, University of Zambia, P.O. Box 32379, Lusaka 10101, Zambia; ladslavm@yahoo.com (L.M.); mudenda68@yahoo.com (B.M.H.); 6 Department of Disease Control, School of Veterinary Medicine, University of Zambia, P.O. Box 32379, Lusaka 10101, Zambia; j.ndebe@yahoo.com (J.N.); esikabala@yahoo.com (E.S.); atakada@czc.hokudai.ac.jp (A.T.); asmweene04@yahoo.com (A.S.M.); 7 Department of Biomedical Sciences, School of Veterinary Medicine, University of Zambia, P.O. Box 32379, Lusaka 10101, Zambia; muleyawalter@gmail.com; 8 Macha Research Trust, Choma 20100, Zambia; 9 Virology Unit, Central Veterinary Research Institute, P.O. Box 33980, Lusaka 10101, Zambia; gdautu@yahoo.co.uk; 10 Ministry of Fisheries and Livestock, P.O. Box 50060, Lusaka 10101, Zambia; 11 Division of Global Epidemiology, Hokkaido University International Institute for Zoonosis Control, North 20, West 10, Kita-ku, Sapporo 001-0020, Hokkaido, Japan; kajihara@czc.hokudai.ac.jp (M.K.); akinam@czc.hokudai.ac.jp (A.M.-K.); 12 Hokudai Center for Zoonosis Control in Zambia, Hokkaido University International Institute for Zoonosis Control, P.O. Box 32379, Lusaka 10101, Zambia; yongjin_qiu@czc.hokudai.ac.jp (Y.Q.); hidea-hi@czc.hokudai.ac.jp (H.H.); 13 Laboratory of Zoonotic Diseases, Faculty of Applied Biological Sciences, Gifu University, Gifu 501-1193, Gifu Prefecture, Japan; naotoito@gifu-u.ac.jp; 14 Division of Collaboration and Education, Hokkaido University International Institute for Zoonosis Control, North 20, West 10, Kita-ku, Sapporo 001-0020, Hokkaido, Japan; czc.sugimoto@gmail.com; 15 Division of Infection and Immunity, Hokkaido University International Institute for Zoonosis Control, North 20, West 10, Kita-ku, Sapporo 001-0020, Hokkaido, Japan

**Keywords:** Africa, antibody titer, domestic dog, immunization coverage, rabies, Zambia

## Abstract

Rabies remains endemic in Zambia. Despite conducting canine vaccinations in Lusaka district, the vaccination coverage and actual seropositivity in the dog population in Lusaka district are rarely evaluated. This study estimated the seropositivity-based immunization coverage in the owned dog population in Lusaka district using the expanded program on immunization cluster survey method. The time-series trend of neutralizing antibodies against rabies in vaccinated dogs was also evaluated. Of 366 dogs in 200 dog-owning households in Lusaka district, blood samples were collected successfully from 251 dogs. In the sampled dogs, 42.2% (106/251) had an antibody titer ≥0.5 IU/mL. When the 115 dogs whose blood was not collected were assumed to be seronegative, the minimum immunization coverage in Lusaka district’s owned dog population was estimated at 29.0% (95% confidence interval: 22.4–35.5). It was also found that a single vaccination with certified vaccines is capable of inducing protective levels of antibodies. In contrast, higher antibody titers were observed in multiple-vaccinated dogs than in single-vaccinated dogs, coupled with the observation of a decline in antibody titer over time. These results suggest the importance of continuous booster immunization to maintain herd immunity and provide useful information to plan mass vaccination against rabies in Zambia.

## 1. Introduction

Rabies is one of the most feared zoonotic diseases and is endemic in more than 100 countries and territories [1]. Approximately 59,000 human deaths occur from rabies annually, mostly in Asian and African countries [2]. As the majority of human rabies is transmitted by dogs [3], vaccination of dogs and the provision of human post-exposure prophylaxis are the most important and efficient control measures for rabies [1,4,5].

Canine rabies vaccination has been conducted to maintain herd immunity in dog populations. It is known that 20–45% of dogs must always be immune to interrupt the rabies transmission in a dog population, and this coverage is recognized as the critical vaccination coverage of rabies [6]. During canine mass vaccination campaigns, which are usually conducted annually in resource-limited countries, it is well understood that a 70% vaccination coverage must be attained in a campaign [1,6,7]. This coverage, which is higher than the abovementioned critical threshold (i.e., 20–45%), is required to prevent the decline of herd immunity below the critical threshold during the intervals between vaccination campaigns [6,8,9]. Particularly in highly dense, large, and connected dog populations, spatial heterogeneity in vaccination coverage allows rabies transmission to be sustained [10,11,12]. Although there is no evidence that rabies virus transmission depends on the dog population density [6,13], epidemics likely continue for longer durations, with more cases in larger and higher-density populations [10]. Therefore, rabies control programs need to include comprehensive canine vaccination across dog populations, particularly in urban settings.

Dog owners’ accessibility to canine rabies vaccines is considered to be better in urban than in rural settings [4,14,15]. Furthermore, dog owners who reside in high-income residential areas are likely to intentionally vaccinate their dogs [16,17]. Therefore, in urban settings, a combination of mass vaccination campaigns in low-income residential areas and vaccination in veterinary clinics in high-income residential areas could effectively enhance and maintain the canine herd immunity against rabies in urban settings [16]. In such situations, household surveys are necessary to assess the vaccination coverage achieved in urban settings because low- and high-income residential areas, which are probably covered by mass vaccination campaigns and owners’ voluntary vaccination, are sometimes intermingled. However, owners’ improper maintenance of vaccination certificates makes an assessment more difficult in household surveys. The World Health Organization (WHO) states that routine serological monitoring after canine mass vaccination campaigns is unnecessary if the following criteria are observed: (1) high-quality vaccines manufactured according to international standards have been used; (2) vaccinators have been trained in the proper administration and handling of vaccines as well as of dogs; and (3) the cold chain has been maintained throughout [1]. However, in cases where vaccination certificates are unavailable, serological evaluation will provide helpful information to assess the actual immunization coverage, defined as the proportion of dogs that retain protective antibody titers in a dog population.

In the Republic of Zambia, rabies is endemic countrywide [18,19,20,21]. Most domestic dogs are usually allowed to roam freely. At the same time, some of them are kept confined to their houses surrounded by fences or brick walls, particularly in the capital city of Lusaka. Rabies control programs have been promoted in Lusaka district, and a considerable number of canine rabies vaccinations have been implemented during mass vaccination campaigns and at veterinary clinics. A household survey conducted in a low-income, densely populated area of Lusaka in the early 1990s demonstrated a canine vaccination coverage of 16% (26/160 dogs) based on the vaccination status [16]. Although mass vaccinations have been conducted in many parts of Lusaka district, particularly in populated residential areas, the vaccination coverage of the domestic dog population in Lusaka district has never been estimated despite the continued presence of rabies in both humans and animals [18,19,22]. Therefore, this study aimed to estimate the “vaccination coverage” based on vaccination certificates and the “actual immunization coverage” based on the seropositivity in the owned dog population of Lusaka district and to retrospectively evaluate antibody decline in vaccinated dogs by measuring antibody titers with reference to the dates of vaccination.

## 2. Materials and Methods

### 2.1. Study Area

**Figure 1:**
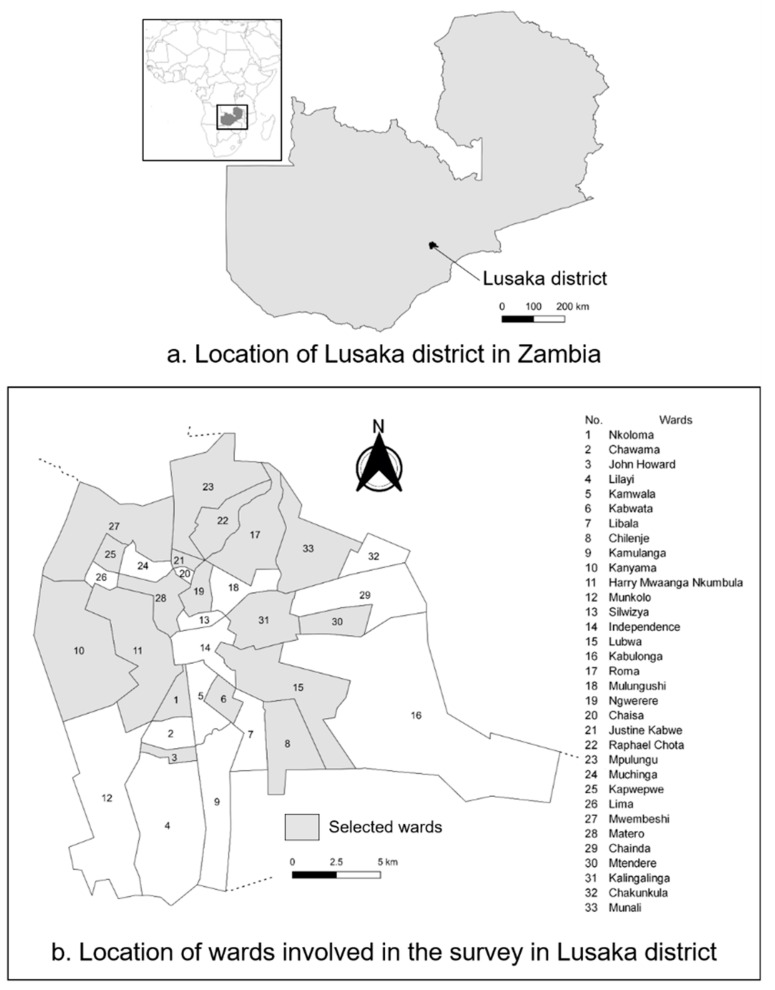
Location of the study area: (**a**) location of Lusaka district in Zambia; (**b**) location of the wards involved in the survey in Lusaka district. The selected wards and corresponding numbers are as follows: (1) Nkoloma, (3) John Howard, (6) Kabwata, (8) Chilenje, (10) Kanyama *, (11) Harry Mwaanga Nkumbula *, (15) Lubwa, (17) Roma, (19) Ngwerere, (21) Justine Kabwe, (22) Raphael Chota, (23) Mpulungu, (25) Kapwepwe, (27) Mwembeshi, (28) Matero, (30) Mtendere, (31) Kalingalinga, and (33) Munali. Asterisks (*) denote the wards where double clusters were selected.

The study was conducted in Lusaka district, located in Lusaka Province, in the central part of Zambia (Figure 1). The district covers 360 km^2^ with a total human population of 1,747,152 individuals according to the 2010 census [23]. The dog population was estimated at 44,054 dogs between 2017 and 2018 by the Department of Veterinary Services, Zambia.

### 2.2. Cluster Survey Method

Sampling was conducted from 23 March 2015 to 17 April 2015 following the expanded program on immunization (EPI) cluster survey, with modification [24,25]. The sampling in this study aimed to estimate the immunization coverage within a ±10% desired precision, with a 95% confidence interval (CI). This survey consisted of a two-stage cluster sampling. In the first stage, 20 of the wards were sampled as clusters with a probability proportionate to the households’ size in the wards (Table S1). The selected clusters are shown in Figure 1. In the second stage, at least ten households that owned dogs were selected within each cluster. The subjects were chosen by selecting a household randomly, and every eligible subject in the household was included in the sampling [26], with a few exceptions mentioned in the next paragraph.

The survey was accompanied by one veterinary assistant officer from the Lusaka district veterinary office. In the dog-owning households selected, the purpose of the study was explained to the head of the household or suitable representatives, and their verbal consent for participation was obtained. All dogs in the households selected were included in the survey for blood sample collection and determination of previous vaccination certificates. However, in situations where the dog was too vicious, could not be restrained by the owner, or was less than 3 months of age, the dog was not sampled. Information on the dog(s), way of keeping dog(s) in the household, previous vaccination, product name of the previous vaccine, manufacturer, lot number, and validity of the vaccination was collected in each household.

This study designated the proportion of dogs that had valid vaccination certificates among the targeted dog population as “vaccination coverage”. To estimate the vaccination coverage based on the information in the previous vaccination certificates, we followed the criteria as follows: (i) the vaccination certificate was valid for 6 months (180 days) and 1 year (365 days) in case of the first vaccination and from the second vaccination, respectively; and (ii) dogs whose vaccination history was unclear without a previous vaccination certificate and dogs whose vaccination certificates had expired were regarded as unvaccinated. These criteria correspond to the “Protocol on Rabies Disease Control in Zambia”, as stipulated in the Control of Dogs Act, Cap 247 of the Laws of Zambia.

### 2.3. Blood Sample Collection from Owned Dogs

Blood samples were collected to measure the neutralizing antibody titers against the rabies virus. This was done to estimate the proportion of seropositive dogs among the targeted dog population, which was defined as the “actual immunization coverage”, and to assess the antibody decline over time in the vaccinated dogs. To estimate the actual immunization coverage, 251 dogs were sampled according to the EPI cluster survey. To assess the antibody decline over time, 27 additional blood samples were collected in the same period from Lusaka district, in addition to the 251 samples. Of the 278 samples, 37 samples were obtained from dogs that had received a single vaccination using Rabisin (Merial, Lyon, France), Rabigen-mono (Virbac, Carros, France), or Rabies Vet (Bio-Med, Ghaziabad, India) before our sampling (Table S2). Similarly, 39 samples were obtained from the dogs that had been vaccinated multiple times with the aforementioned rabies vaccine products before our sampling (Table S3). This information was obtained from vaccination certificates.

The cephalic vein on the foreleg was used to collect blood. Briefly, 2 mL of blood from each dog was collected in sterile tubes and allowed to settle at room temperature for 1 h to promote coagulation. Blood samples were subsequently stored at 4 °C overnight to exude serum. Afterward, sera were collected into new tubes, and the samples were stored at −80 °C until being shipped to Japan for subsequent laboratory analyses. According to the Protocol on Rabies Disease Control in Zambia, puppies aged below 3 months were not sampled as they are ineligible for the rabies vaccination; unhealthy dogs were also not sampled.

### 2.4. Measurement of Antibody Titer Against Rabies

Antibodies against the rabies virus in the serum samples were measured using the fluorescent antibody virus neutralization (FAVN) test at the Hokkaido University International Institute for Zoonosis Control, Japan, according to the Manual of Diagnostic Tests and Vaccines for Terrestrial Animals 2013 [27] released by the World Organisation for Animal Health (OIE). Briefly, the rabies virus challenge virus standard (CVS) strain and BHK-21 C13 cells (ATCC CCL-10) were used for the FAVN test. The serum samples were first heat-treated at 56 °C for 30 min to inactivate the complements and serially diluted in 96-well plates. The diluted serum samples were incubated with 100 TCID_50_ (50% tissue culture infective dose) of CVS in 50 µL. Any un-neutralized CVS could replicate on BHK-21 C13 cells and be detected by fixation with 10% formalin and staining with fluorescein isothiocyanate anti-rabies monoclonal globulin (Fujirebio Diagnostics, Malvern, PA, USA). The stained cells were evaluated qualitatively by fluorescent microscopy. The Spearman–Kärber method [28] was used to calculate the 50% endpoint titers of the serum, and the titers were converted into international units (IU/mL) by comparison with the OIE-positive standard serum (ANSES, Maisons-Alfort, France) with a known neutralizing titer. Following the WHO recommendations, a neutralizing antibody titer ≥0.5 IU/mL was regarded as positive [29,30], which is a criterion required for international dog movement [31]. Furthermore, another threshold of 0.2 IU/mL was adopted as the “minimum” titer that was considered adequate to protect host dogs from the rabies virus infection, which was studied by Bunn et al. between 1983 and 1984 (cited in Aubert 1992 [32]). For every sample whose titer could not be measured at a certain value because of the limit of detection, particularly in the range less than 0.1 IU/mL, we assigned an arbitrary value corresponding to the maximum possible value to be able to perform the analysis, for example, ≤0.042, ≤0.056, ≤0.073, and ≤0.096 IU/mL of the actual detected values were regarded as 0.042, 0.056, 0.073, and 0.096 IU/mL, respectively.

### 2.5. Data and Statistical Analyses

Excel 2016 was used for data input. Subsequently, the vaccination and actual immunization coverage according to the results of the FAVN test were calculated. Data analyses of the antibody titers were performed using R version 3.6.3 [33].

The association between the “vaccination status”, represented by the validity of the vaccination certificate, and “seropositivity”, represented by antibody retention at thresholds of ≥0.5 IU/mL, and the association between the vaccination status/seropositivity and dog sex were tested using Fisher’s exact test and the R package “fmsb” in R version 3.6.3. A *p*-value < 0.05 was considered statistically significant. The *p*-values in the multiple tests were adjusted using the Benjamini–Hochberg method [34].

## 3. Results

### 3.1. Dog Population Characteristics

Two hundred dog-owning households were selected for participation in this study. The mean number of dogs per dog-owning household was 1.8 (median 1, minimum 1, and maximum 7). Of the 366 dogs owned in the surveyed households, blood samples were collected successfully from 251 dogs for the EPI cluster survey. The male-to-female ratio in the sampled dogs was 1.04:1. The mean age of the sampled dogs was 1.2 years (median: 1.3 years). The age distribution of the sampled dogs is shown in Table 1. A total of 62.9% of the sampled dogs (158/251) were allowed to roam freely, 22.3% (56/251) were kept as free-range only within the fenced premises according to the owners’ reports, 4.4% (11/251) were reported to be confined in cages or kept by chains, and 10.4% (26/251) were reported to be kept using a mixed style of free-range inside the premises and confinement, depending on the time and situation.

### 3.2. Rabies Vaccination and Immunization Coverage in Dogs

A total of 19.9% of the sampled dogs (50/251) had valid vaccination certificates (Table 2). In contrast to this certificate-based vaccination coverage, 42.2% (106/251) had sufficiently high levels of rabies virus-neutralizing antibodies (i.e., ≥0.5 IU/mL) (Table 2a). When a value of 0.2 IU/mL was adopted as the threshold titer, 52.6% (132/251) had the minimum protective levels of the antibodies at the sampling time (Table 2b). For a conservative estimate of the vaccination coverage among the entire owned dog population in Lusaka district, the 115 dogs excluded from the study were added to the denominator, with the assumption that all of them had never been vaccinated; minimum vaccination coverage of 13.7% (50/366; 95% CI: 8.7–18.6) was obtained for the owned dog population in Lusaka district based on the EPI cluster survey estimates. In the same manner, minimum immunization coverage, defined as the minimum proportion of seropositive dogs among the total owned dog population in Lusaka district, was also estimated, and the results are presented in Table 3. The geometric mean titer (GMT) of 251 serum samples was 0.43 IU/mL (95% CI: 0.33–0.55; minimum: 0.042 IU/mL; median: 0.22 IU/mL; maximum: 159.9 IU/mL).

Dogs that had valid vaccination certificates were significantly seropositive, with a 0.5 IU/mL threshold titer, compared with dogs whose status was uncertain/expired or had never been vaccinated (*p*-values < 0.01). Dog sex was neither associated with vaccination status (*p*-values > 0.5) nor seropositivity with 0.5 IU/mL of the threshold (*p* = 0.16).

### 3.3. Antibody Decline in Vaccinated Dogs

**Figure 2:**
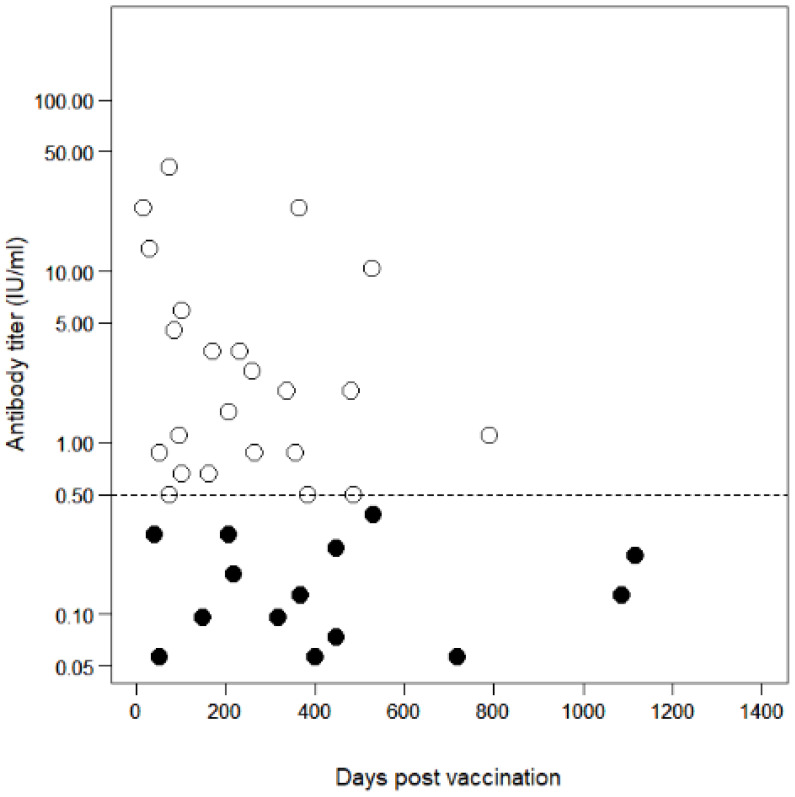
Antibody decline in single-vaccinated dogs (*n* = 37). Open circles represent samples that had antibody titers ≥0.5 IU/mL. Filled circles represent samples that had antibody titers <0.5 IU/mL. The dashed line shows the antibody titer’s threshold level (0.5 IU/mL) required for international dog movement.

The association of antibody titers in single-vaccinated dogs (*n* = 37) with days post vaccination (dpv) is presented in Figure 2. In the tested dogs, the probabilities of vaccination success within 180 dpv, applying the thresholds of 0.5 and 0.2 IU/mL were 78.6% (95% CI: 49.2–95.3; *n* = 14) and 85.7% (95% CI: 57.2–98.2), respectively. The GMT among the single-vaccinated dogs (*n* = 37; minimum dpv: 18; maximum dpv: 1117) was 0.81 IU/mL (95% CI: 0.44–1.48), whereas the GMT in those within 180 dpv (*n* = 14) was 1.53 IU/mL (95% CI: 0.49–4.79). All data for each sample regarding the antibody titer, dpv, vaccine product, date of blood sampling, and date of the last vaccination are provided in Table S2.

**Figure 3:**
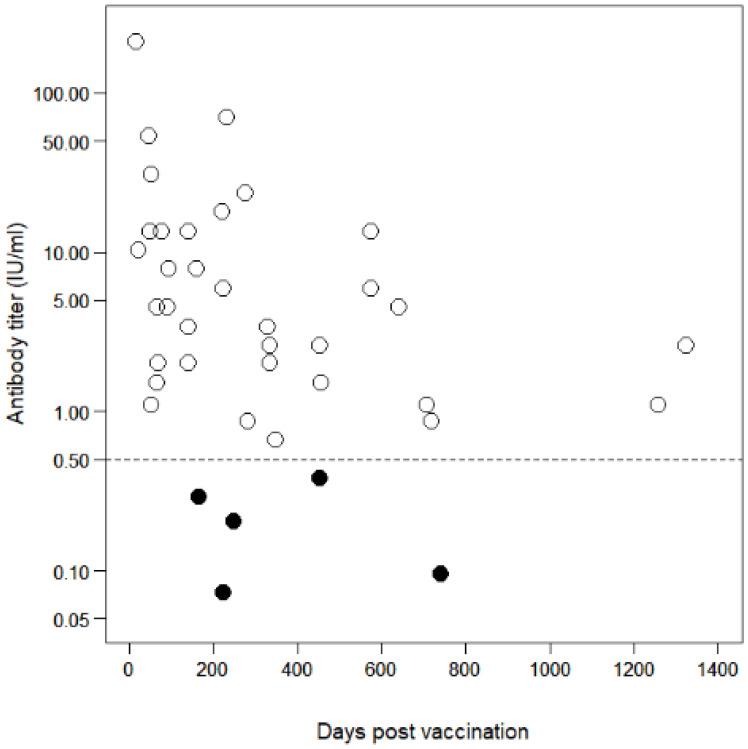
Antibody decline in multiple-vaccinated dogs (*n* = 39). Open circles represent samples that had antibody titers ≥0.5 IU/mL. Filled circles represent samples that had antibody titers <0.5 IU/mL. The dashed line shows the antibody titer’s threshold level (0.5 IU/mL) required for international dog movement.

The time-series trend of antibody titer in multiple-vaccinated dogs (*n* = 39) is presented in Figure 3. In these dogs, the probabilities of vaccination success within 365 dpv, with the thresholds of 0.5 and 0.2 IU/mL, were 89.3% (95% CI: 71.8–97.7; *n* = 28) and 96.4% (95% CI: 81.7–99.9), respectively. The GMT among multiple-vaccinated dogs (*n* = 39; minimum dpv: 18; maximum dpv: 1323) was 3.34 IU/mL (95% CI: 1.90–5.86), whereas the GMT in those within 365 dpv (*n* = 28) was 4.49 IU/mL (95% CI: 2.23–9.03). All data for each dog regarding the antibody titer, dpv, number of vaccinations, vaccine product, and date of sampling are provided in Table S3.

## 4. Discussion

This study estimated the immunization coverage and demonstrated the antibody decline over time in vaccinated dogs in Lusaka district of Zambia. This is the first report describing the actual immunization coverage against rabies, represented by a proportion of seropositive dogs in the owned dog population in the capital city of Lusaka, Zambia.

Even though vaccination certificates had expired or were uncertain in nearly half of the dogs (119/251), over 50% of such dogs (62/119) had rabies virus-neutralizing antibodies with titers ≥0.5 IU/mL. Over 60% of those (77/119) dogs had antibody titers ≥0.2 IU/mL (Table 2). Therefore, the immunization coverage, defined as the proportion of dogs that had actual protective levels of the antibody, was not extremely low as a whole, even though one-third of the dogs had never been vaccinated, based on their owners’ statements. The measurement of antibody titer is unnecessary to evaluate immunization coverage after a mass vaccination campaign if certified vaccines are used and the vaccinators are well trained to conduct the vaccination [1]. However, it is difficult to assess the immunization coverage if owners do not properly preserve the vaccination certificates. Indeed, 14.6% of the dogs (12/82 dogs that had never been vaccinated) in this study had antibody titers ≥0.2 IU/mL, although they were declared as never been vaccinated by their owners. However, it should be noted that these antibodies against the rabies virus may come from nonlethal exposure to antigens, for instance, through the consumption of carcasses that have died of rabies or another lyssavirus infection [35,36,37]. As one-third of dogs could not be designated as vaccinated or not, the further necessity of improving dog-owner responsibility, such as good conduct of vaccination and proper preservation of vaccination certificates, is emphasized to enhance rabies control in dogs. Regarding the level of herd immunity, the immunization coverage was 52.6% among the dogs tested and the minimum immunization coverage was estimated at 36.1% in Lusaka district, when 0.2 IU/mL of titer was adopted as the threshold. These values would be moderately sufficient to protect the dog population from a rabies outbreak compared to the critical vaccination coverage of 20–45% that is required to interrupt rabies transmission in a dog population [6]. However, it should be noted that the immunization coverage demonstrated in this study targeted the owned dog population in Lusaka district without involving the ownerless dog population. It should be reminded that herd immunity needs to be maintained in the total dog population, including both owned and ownerless dogs, although the ownerless dog population in Lusaka district seemed to be very low [16], in addition to the increasing evidence that most free-roaming dogs in rabies-endemic countries are owned [9,38,39,40].

The ordinary vaccination coverage observed in this study was lower than that in earlier studies conducted in other rabies-endemic countries, such as 85% in Santa Cruz de la Sierra, Bolivia [41], and 70% in Thungsong district, Thailand [42]. Alternatively, the actual immunization coverage observed in this study was similar to or slightly less than the seropositivity-based immunization coverage, with 0.5 IU/mL of threshold titer recorded in other African countries, such as a 42.6% immunization coverage in Ilorin city, Nigeria, by stratified random sampling [17]. That earlier study mentioned both a lack of stable rabies vaccination programs in the city and vaccination failure that were common in Nigeria [43,44] as factors contributing to the immunization coverage observed [17]. In Gaborone, Botswana, a 54% seropositivity in dogs sampled in animal clinics was reported [45]. Moreover, in Emalahleni in the Eastern Cape Province of South Africa, immunization coverage of 32% was reported, with vaccination coverage of 56% among a randomly sampled dog population [46].

The ordinary immunization/vaccination coverage in African urban settings described above is remarkably higher than the ordinary vaccination coverage of below 10% recorded previously in rural Zambia [38,47]. This could be attributed to the differences in urban and rural settings regarding the availability of vaccine products and the dog owners’ accessibility to the vaccine, affordability of the canine rabies vaccine, and so on [15]. As demonstrated in rabies-endemic African countries, free rabies mass vaccination campaigns are capable of achieving the WHO-recommended vaccination coverage of 70% [1,7], whereas owner-charged vaccination campaigns achieve a vaccination coverage that is insufficient to prevent the transmission of rabies [40,48,49]. Although dog owners, particularly in rural settings, need free rabies mass vaccination to achieve 70% vaccination coverage in a campaign, a certain number of dog owners in urban settings may be capable of paying for regular canine vaccination. Therefore, it is possible to maintain the critical threshold coverage in urban settings with a combination of mass vaccination campaigns and veterinary clinic-based vaccination unless the supply of high-quality vaccine products is unstable. As there is a tendency for higher seropositivity in high-income residential areas and lower seropositivity in low-income residential areas in urban settings [17], differences in the owners’ income level and the affordability of the canine rabies vaccine could be factors influencing the immunization coverage and heterogeneity in the dog population in urban settings, where the residents’ characteristics may be more varied than those in rural areas. This study did not analyze the differences in vaccination coverage and actual immunization coverage among the selected wards by the income level of the dog owners. However, this should be considered when making policies aimed at improving vaccination coverage with a combination of owner-charged rabies vaccination and free rabies mass vaccination to raise the vaccination coverage in the entire city of Lusaka.

This study demonstrated that the antibody declines over time among vaccinated dogs in Lusaka district, Zambia. It retrospectively verified that a single vaccination with certified vaccines could have acceptably induced and retained protective antibodies for at least 180 dpv, as the certificate for the first vaccination is regarded as valid for 180 days. However, the peak titer has been reported to influence prolonged antibody retention after vaccination [50]. The higher the peak titer, the longer the antibody titer remains potent enough to protect the host animal [50]. In contrast, if the peak titer is low, the antibody titer will decline to below the protective level even within the period of vaccination validity [50]. This highlights the possibility of the rapid decline in antibody titers among the dog population studied even if they had a protective level of antibody titers at the time of sampling because the titers, which would be considered peak titers 3–6 weeks after vaccination [1,50,51,52], were not high in some individuals in this study. Here only three dogs retrospectively corresponded to the duration approximately 3–6 weeks after the first vaccination. Antibody titers for these dogs were 23.4, 13.5, and 0.29 IU/mL after 18, 32, and 42 dpv, respectively. A field trial showed a GMT of 14.8 IU/mL as a peak titer at 30 dpv, which declined to 0.81 IU/mL at 180 dpv in a rabies-endemic African country [50]. We could not determine whether the aforementioned titers observed between 3 and 6 weeks after vaccination would be retained at the level of ≥0.2 or ≥0.5 IU/mL until 180 dpv, because we did not prospectively assess the kinetics of the antibody titers in individuals. However, it should be emphasized that declines in individual antibody titers must be considered during the planning of rabies mass vaccinations with the aim of maintaining herd immunity.

Nevertheless, the probability of vaccination success was 78.6% in the single-vaccinated dogs and 89.3% in the multiple-vaccinated dogs with the threshold of 0.5 IU/mL, and 85.7% in the single-vaccinated dogs and 96.4% in the multiple-vaccinated dogs with the threshold of 0.2 IU/mL. Other field studies demonstrated a seroconversion of 83% in field dogs in South Africa [46] and 87.2–93.7% seroconversion and antibody retention at ≥0.5 IU/mL until 180 dpv from single-vaccinated dogs in Sri Lanka [52], both using commercial rabies vaccine products. Our findings are similar to those reported in these abovementioned studies, although our evaluation was performed retrospectively. As demonstrated previously, multiple vaccinations (boosters) enhance seroconversion and induce long-lasting antibody retention [51,52]. In this study, dogs that received multiple vaccinations had a higher GMT and a higher probability of vaccination success than those observed in the single-vaccinated dogs.

This study observed a certain proportion of seronegative dogs among vaccinated dogs despite their valid vaccination certificates. This fact suggests two situations. First, there is a possibility that those seronegative dogs had seroconverted once after the vaccination and, subsequently, the antibody titer decreased below the threshold titer by the date of sampling. The second possible situation is that the seronegative dogs had truly never seroconverted after the vaccination at the time of sampling. Although the reasons for the presence of seronegative dogs, despite a valid vaccination status, remain unclear in this study, it should be considered that a certain proportion of dogs will not seroconvert in a mass vaccination campaign. This is important to note when calculating the desired target vaccination coverage during the planning phase of the mass vaccination campaigns. The reasons for vaccine failure may be various factors, such as a break in the cold chain, inadequate vaccination technique, or host animal factors.

Regarding a break in the cold chain, the Nobivac Rabies vaccine (Merck Animal Health, Madison, NJ, USA), one of the high-quality commercially inactivated canine rabies vaccines, is thermotolerant [53]. Power loss occurs in Lusaka district from time to time; however, information on the thermotolerance of Rabisin, a commercially inactivated vaccine used commonly in Lusaka district, is lacking. Furthermore, another earlier study demonstrated that a vast majority of dogs in endemic rabies countries seroconverted successfully (with the threshold of 0.5 IU/mL), regardless of health status. However, there were substantial variations in titers that arose partly from clinical conditions and lactation at vaccination [50]. The study, being cross-sectional and retrospective in nature, did not analyze the association between the seroconversion or level of antibody titer and the health status or lactation at the time of vaccination. However, this may be another concern for seroconversion and the introduction of a long-lasting antibody titer.

This study presented the findings that help understand the current achievements and situations of rabies control programs in Lusaka. The data presented in this study have great potential to guide the planning and implementation of rabies vaccination programs in Lusaka city and contribute positively to achieving the global goal of “Zero by 30”.

## 5. Conclusions

This study demonstrated the vaccination coverage and actual immunization coverage in the owned dog population in Lusaka district, Zambia. Although the vaccination coverage estimated based on vaccination certificates’ validity was low, the actual immunization coverage was moderately acceptable to confer herd immunity against rabies. This discordance was attributed to owners’ improper storage of vaccination certificates for their dogs. Therefore, it is important to continue providing information and education on responsible dog ownership to dog owners to promote effective rabies control in dogs in Lusaka and Zambia. This study further verified that a single vaccination with certified vaccines could induce protective antibodies up to 180 dpv; however, regular boosters are necessary to enhance and maintain protective antibody titers and improve herd immunity. The data presented in this study will further strengthen the execution of rabies control programs in Zambia and other rabies-endemic countries and contribute to achieving the goal of the “Zero by 30” global strategic plan for rabies control.

## Figures and Tables

**Table 1:** Age distribution of the dogs involved in the EPI cluster survey.

Male	Age (Months)	Female
15	3–11	23
17	12–23	22
11	24–35	10
18	36–47	12
9	48–59	9
16	60–71	12
5	72–83	4
5	84–95	2
8	Over 96	0
24	Unidentified	29
128	Total	123

**Table 2:** Validity of the vaccination status and seropositivity.

a. Seropositivity with a threshold of 0.5 IU/mL					
	Valid	Uncertain	Expired	Never Vaccinated Before	Total
Seropositive	40	38	24	4	106 (42.2)
Seronegative	10	34	23	78	145 (57.8)
Total	50 (19.9)	72 (28.7)	47 (18.7)	82 (32.7)	251
Values in parentheses are the proportion of the corresponding status (%).					
**b. Seropositivity with a threshold of 0.2 IU/mL**					
	**Valid**	**Uncertain**	**Expired**	**Never Vaccinated Before**	**Total**
Seropositive	43	45	32	12	132 (52.6)
Seronegative	7	27	15	70	119 (47.4)
Total	50 (19.9)	72 (28.7)	47 (18.7)	82 (32.7)	251
Values in parentheses are the proportion of the corresponding status (%).					

**Table 3:** Immunization coverage (proportion of dogs that had actual antibodies against rabies).

	Immunization Coverage (*n* = 251)		Minimum Immunization Coverage (*n* = 366) ^†^	
	Threshold: 0.5 IU/mL	Threshold: 0.2 IU/mL	Threshold: 0.5 IU/mL	Threshold: 0.2 IU/mL
Coverage (%)	42.2 (33.6–50.9)	52.6 (43.9–61.3)	29.0 (22.4–35.5)	36.1 (29.1–43.0)
Values in parentheses are obtained at 95% confidence intervals.				

† Including 115 dogs excluded from blood sampling, assuming that they were seronegative.

## Data Availability

The data presented in this study are available in Supplementary Martials.

## Supplementary Materials

The following are available online at https://www.mdpi.com/article/10.3390/pathogens10060738/s1, Table S1: Selected wards in the EPI cluster survey; Table S2: Vaccination history of single-vaccinated dogs; Table S3: Vaccination history of multiple-vaccinated dogs.
